# A Serological Survey of Infectious Disease in Yellowstone National Park’s Canid Community

**DOI:** 10.1371/journal.pone.0007042

**Published:** 2009-09-16

**Authors:** Emily S. Almberg, L. David Mech, Douglas W. Smith, Jennifer W. Sheldon, Robert L. Crabtree

**Affiliations:** 1 Department of Natural Resources, Science, and Management, University of Minnesota, St. Paul, Minnesota, United States of America; 2 USGS Northern Prairie Wildlife Research Center, St. Paul, Minnesota, United States of America; 3 Yellowstone Center for Resources, Wolf Project, Yellowstone National Park, Wyoming, United States of America; 4 Yellowstone Ecological Research Center, Bozeman, Montana, United States of America; University of Georgia, United States of America

## Abstract

**Background:**

Gray wolves (*Canis lupus*) were reintroduced into Yellowstone National Park (YNP) after a >70 year absence, and as part of recovery efforts, the population has been closely monitored. In 1999 and 2005, pup survival was significantly reduced, suggestive of disease outbreaks.

**Methodology/Principal Findings:**

We analyzed sympatric wolf, coyote (*Canis latrans*), and red fox (*Vulpes vulpes*) serologic data from YNP, spanning 1991–2007, to identify long-term patterns of pathogen exposure, identify associated risk factors, and examine evidence for disease-induced mortality among wolves for which there were survival data. We found high, constant exposure to canine parvovirus (wolf seroprevalence: 100%; coyote: 94%), canine adenovirus-1 (wolf pups [0.5–0.9 yr]: 91%, adults [≥1 yr]: 96%; coyote juveniles [0.5–1.5 yrs]: 18%, adults [≥1.6 yrs]: 83%), and canine herpesvirus (wolf: 87%; coyote juveniles: 23%, young adults [1.6–4.9 yrs]: 51%, old adults [≥5 yrs]: 87%) suggesting that these pathogens were enzootic within YNP wolves and coyotes. An average of 50% of wolves exhibited exposure to the protozoan parasite, *Neospora caninum*, although individuals’ odds of exposure tended to increase with age and was temporally variable. Wolf, coyote, and fox exposure to canine distemper virus (CDV) was temporally variable, with evidence for distinct multi-host outbreaks in 1999 and 2005, and perhaps a smaller, isolated outbreak among wolves in the interior of YNP in 2002. The years of high wolf-pup mortality in 1999 and 2005 in the northern region of the park were correlated with peaks in CDV seroprevalence, suggesting that CDV contributed to the observed mortality.

**Conclusions/Significance:**

Of the pathogens we examined, none appear to jeopardize the long-term population of canids in YNP. However, CDV appears capable of causing short-term population declines. Additional information on how and where CDV is maintained and the frequency with which future epizootics might be expected might be useful for future management of the Northern Rocky Mountain wolf population.

## Introduction

Several high-mortality disease outbreaks among carnivore populations have demonstrated the potential for pathogen-induced population declines [1]–[6] and have emphasized the role of infectious disease in carnivore conservation [7], [8]. These outbreaks have underscored both the need for better baseline data on disease prevalence, and a better understanding of the ecology of disease in wild populations [7], [9].

Yellowstone National Park (YNP) is home to one of the largest, protected, intact suites of carnivores in the contiguous United States. Gray wolves (*Canis lupus*) were reintroduced into the Yellowstone Ecosystem after a >70 year absence, and as part of recovery efforts, the population is closely monitored [10], [11]. In 1999 and 2005, pup survival was significantly reduced, suggestive of a disease outbreak [12].

Thus we sought to use long-term serological data to identify temporal, spatial, and demographic patterns of pathogen exposure among wolves, coyotes (*Canis latrans*), and foxes (*Vulpes vulpes*) in YNP. We screened for exposure to canine parvovirus (CPV), canine adenovirus type-1 (CAV-1), canine distemper virus (CDV), and canine herpesvirus (CHV), all of which can inflict morbidity and mortality in canids (Table S1) [13]–[19]. In wolves, we also screened for exposure to *Neospora caninum*, a protozoan parasite whose life cycle includes canids, the definitive hosts where sexual reproduction takes place, and ungulates, the intermediate hosts [20], [21]. *N. caninum* is transmitted between canids and ungulates when a canid consumes infected ungulate tissue. *N. caninum* reproduces in the canid’s intestines, and oocysts are shed in feces and then consumed by ungulates through contaminated forage and water. *N. caninum* infection can cause high rates of abortion in cattle, and thus is a pathogen of special interest to the local ranching community.

We assessed whether each of the pathogens of interest were enzootic or epizootic in the YNP canid community, and whether pathogen exposure varied by region of the park in relation to canid density. We investigated if behavioral differences between resident and transient coyotes, the latter potentially interacting with many more individuals across many different packs, and thus potentially at greater risk for pathogen exposure, contributed to differences in exposure risk. Host age was used primarily to examine temporal patterns of exposure, but it was also evaluated as a risk factor for recent or current infection with CHV and *N. caninum*.

Survival data were not available for coyotes or foxes. However, we did examine the relationship between pathogen exposure and wolf-pup survival. Furthermore, we used comparisons of exposure patterns among the canids to assess the likelihood of single versus multi-host pathogen transmission within YNP.

## Materials and Methods

### Ethics statement

All wolves, coyotes, and foxes used in this study were handled in strict accordance with recommendations from the American Society of Mammalogists [22], [23], and all animal work was approved by a National Park Service veterinarian, a YNP review committee, and by the YNP superintendent.

### Study area

YNP encompasses 8,991-km^2^ of protected land in northwestern Wyoming and adjacent parts of Montana and Idaho in the western United States (44°33′ N, 110°30′ W). YNP is surrounded by the Greater Yellowstone Ecosystem (GYE), a 60,000-km^2^ area that includes Yellowstone and Grand Teton National Parks, national forests, wildlife refuges, and a mosaic of state and private lands. YNP is mountainous (elevation range: 1,500 to 3,800 m), and its steep gradients in elevation, soil, and climate contribute to varied land cover, including riparian vegetation, shrubland, grassland, alpine meadows, and mixed coniferous forests.

We divided the park into two units, the Northern Range (NR) and the Interior, based on ecological and physiographical differences [24]. The 1,000-km^2^ area of the NR within YNP is characterized by lower elevations (1,500–2,200 m), serves as prime wintering habitat for the park’s ungulates [25], and supports a higher density of wolves than the Interior (20–99 wolves/1000 km^2^ versus 2–11 wolves/1000 km^2^ [26]; minimum population count for entire YNP ranged between 118 and 172 wolves between 2000 and 2007 [27]). The Interior of the park (7,991 km^2^) is higher in elevation (>2,500 m), receives higher annual snowfall, and generally supports lower densities of wolves and ungulates, with the exception of a large migratory herd of bison (*Bison bison*).

YNP has an intact suite of terrestrial carnivores, including gray wolves, grizzly (*Ursus arctos)* and black bears (*Ursus americanus*), cougars, coyotes, red foxes, badgers (*Taxidea taxus*), river otters (*Lontra canadensis*), American martens (*Martes americana*), short (*Mustela erminea*) and long-tailed weasels (*Mustela frenata*), striped skunks (*Mephitis mephitis*), and wolverines (*Gulo gulo*) [28]. Although extremely rare inside YNP, raccoons (*Procyon lotor*) are present in the surrounding GYE.

### Sample collection

#### Wolves

Since wolf reintroduction to YNP, the National Park Service has captured and radio-collared an annual average of 26 wolves (range 16–38) spanning all known packs in the park (mean packs sampled per year  = 8, range = 4–12). Collaring efforts have generally targeted breeders and ∼50% of each year’s young, with an emphasis on maintaining contact with each pack. We darted wolves from a helicopter during November-March and anesthetized them using a 10 mg/kg dose of Telazol® (tiletamine & zolazepam). We fitted them with radio-collars (Telonics, Inc. Mesa, AZ), drew 6–8 ml of blood from the saphenous vein, and categorized the animals as pups (<12 months) or adults, with precise ages estimated from tooth wear [29]. We stored whole blood and serum (serum collected by centrifuging whole blood for 15 minutes after 30 minutes of rest) at −80 C until analysis. Following capture, each wolf was identified as belonging to a particular pack.

#### Coyotes and foxes

Staff from the Yellowstone Ecological Research Center (Bozeman, MT, USA) captured coyotes on the NR of YNP during three, multi-year sampling intervals (1991–1992, 1996–1999, and 2003–2005). Foxes were also captured on the NR of YNP, but trapping efforts were less intense and less frequent (1993, 1996, 2003, and 2005). Coyotes and foxes were captured from three regions (Lamar Valley, Blacktail Plateau, and Gardiner River Basin) spanning east to west on the NR inside YNP from September through October. Juvenile and adult coyotes and foxes were captured using padded, offset, center-swivel, foot-hold traps (Victor soft-catch, Woodstream Corp., Lititz, PA, USA) baited with species-specific urine lures. Sex, weight, condition, dentition, and body measurement data were collected for each animal. Individuals were classified as juveniles (0.5–1.5 yrs), young adults (1.6–4.9 yrs), or old adults (≥5 yrs) based on tooth wear [30]. Technicians drew blood and isolated serum as described for wolves and radio-collared (Advanced Telemetry Systems, Isanti, MN, USA; Telonics, Mesa, AZ, USA) the animals.

Monitoring of radio-collared coyotes permitted classifying individuals as residents (i.e., member of a territorial pack) or transients (i.e., solitary individuals, typically inhabiting an area overlapping one or more pack territories). However, we did not have detailed information on individual coyotes’ pack membership or territory location. Thus, exposure data from resident and transient individuals captured in the same region were assumed to be non-independent.

### Serological screening

Sera from wolves (*n* = 239 samples from 220 individuals [94 females, 126 males], during 1997–2007), coyotes (*n* = 110 samples from 109 individuals [44 females, 64 males, 1 unk.], during 1991–1992, 1996–1999, and 2003–2005), and foxes (*n* = 9 samples [3 females, 3 males, 3 unk.] during 1993, 1996, 2003, and 2005) were screened for antibodies to CPV, CAV-1, CDV, CHV, and *N. caninum* (wolf samples only due to insufficient quantities of coyote and fox sera) by the New York State Animal Health Diagnostic Center (Ithaca, NY, USA). Serum neutralization tests [31] were used to detect CAV-1 (positive titer: ≥8), CHV (positive titer: ≥8), and CDV (positive titer: >12) antibodies (titer cutoff selected so as to minimize false positives; data not shown) [32]. A hemagglutination inhibition test was used to detect CPV antibodies (positive titer: ≥20) [33], and an indirect fluorescent antibody test was used to detect *N. caninum* antibodies (positive titer: ≥50) [34], [35].

Data from wolf and coyote pups were used only for animals ≥5 months old to avoid the influence of maternal antibodies [36]–[38]. Repeat samples from the same individual were excluded from the statistical analysis unless they seroconverted or tested negative for two consecutive sampling periods for a given pathogen.

### Wolf-pup survival

We identified wolf dens by tracking radio-collared adult females throughout April. Dens were monitored and pups counted weekly in May and June. Pup counts in the remote Interior were primarily conducted from airplanes. Aerial monitoring of NR dens was often supplemented with ground counts using spotting scopes. We estimated pups born per pack based on high counts observed between May-June. We also estimated pup survival per pack between May and December by calculating the proportion of pups in a pack still alive at the end of December based on weekly (at minimum) aerial and ground counts. Survival data were not available for coyote-pups and fox-kits.

### Analytical and statistical methods

To accommodate the available datasets and the biological differences between both the canid hosts and the pathogens, our analyses involved several different approaches outlined below.

#### Age effects

The viral pathogens CPV, CDV, and CAV-1 generally produce long-lasting immunity in their hosts [14]–[18], so we assumed that once a wolf, coyote, or fox was exposed to one of these pathogens they remained seropositive for life (although Mech and Goyal [unpublished] have found exceptions to this for CPV among wolves). Under this assumption, the serological status of pups, as compared to adults, offers the most precise information about whether a pathogen is circulating in a given year or region. Therefore, we examined wolf-pup and coyote-juvenile data separately from wolf adult (≥1 yr) and coyote adult (≥1.6 yrs) data in the analysis of CPV, CDV, and CAV-1 serological data.

By contrast, CHV, a herpesvirus, produces life-long infections characterized by periods of latency where the virus is present but does not provoke a strong immune response [39]. A negative CHV test result most likely reflects an uninfected individual, although a latent infection cannot be ruled out, whereas a positive result suggests exposure, a more recent infection, or recrudescence [19], [40].

Canids acquire *N. caninum* infections by consuming ungulate tissue infected with the asexual stage of the parasite [41], and a positive *N. caninum* test suggests an active or recent infection with the parasite [35]. Because neither CHV nor *N. caninum* induce consistent, long-term immunity, and because positive results suggest a recent or active infection, we evaluated age class (juvenile (wolf: 0.5–1.9 yrs; coyote: 0.5–1.5 yrs), young adult (wolf: 2–4.9 yrs; coyote: 1.6–4.9 yrs), and old adult (wolf & coyote: ≥5 yrs)) for both wolves and coyotes as a risk factor for recent infection in our analyses of these two pathogens.

#### Temporal, spatial, and demographic patterns of pathogen exposure

Positive and negative test results were analyzed using a logistic, generalized, linear, mixed model with random “pack,” or in the case of coyotes, “region” effects [42], [43]. These random effects were considered important because they allowed for the non-independence of individuals sampled from the same pack or trapping region. We developed sets of *a priori* candidate models including factors such as year, spatial location (wolves only; NR versus Interior), resident versus transient status (coyotes only), and age class (CHV and *N. caninum* analyses only), thought to potentially influence the probability of pathogen exposure (Table 1, Table 2). Year effects were evaluated to test the evidence for temporal variation in exposure, location effects to determine whether NR wolves, living at higher densities, exhibited a higher risk of exposure compared to Interior wolves, and a year*location interaction to allow for the possibility that pathogens circulate at different times between the NR and Interior regions of the park. Among coyotes, we asked whether behavioral differences between residents and transients might contribute to differences in their risk of infection. Finally, as described above, we evaluated age class as a risk factor for recent infection with CHV or *N. caninum*.

**Table 1:** Risk factors evaluated in the analysis of canid pathogen exposure in Yellowstone National Park, 1991–2007.

Factor	Species	Number of categories	Categories	Model notation
Year	Wolves	11	1997–2007	Year
	Coyotes	9	1991–1992, 1996–1999, 2003–2005	Year
Location	Wolves	2	NR, Interior	Location
Resident status	Coyotes	2	Resident, Transient	Resident
Age class*	Wolves	3	Juvenile (0.5–1.9 yrs), Young Adult (2–4.9 yrs), Old Adult (≥5 yrs)	Age Class
	Coyotes	3	Juvenile (0.5–1.5 yrs), Young Adult (1.6–4.9 yrs), Old Adult (≥5 yrs)	Age Class

* Age class was used as a factor in the analysis of canine herpesvirus and *N. caninum* exposure in wolves and coyotes. For all other pathogens, wolf-pup (0.5–0.9 yr) and adult (≥1 yr) and coyote juvenile (0.5–1.5 yrs) and adult (≥1.6 yrs) data were analyzed separately.

Note the differences in factors and categories considered in the analysis of wolf and coyote data. Factors considered in the analysis of exposure to a particular pathogen are detailed in the text.

**Table 2:** *A priori* models of risk factors for canid pathogen exposure in Yellowstone National Park, 1997–2007.

*A priori* model set	
Wolf	Coyote
Intercept	Intercept
Year	Year
Location	Resident
Year + Location	Year + Resident
Year + Location + Year*Location	
Age Class§	Age Class§
Age Class + Year§	Age Class + Year§
Age Class + Location§	Age Class + Resident§
Age Class + Year + Location§	Age Class + Year + Resident§

§ Denotes the additional models considered in the analysis of canine herpesvirus and *Neospora caninum*.

Additive effects are expressed with a plus sign, and interactions between factors are connected with an asterisk.

Sets of candidate models for wolves and coyotes were evaluated for each pathogen using model-selection procedures based on Akaike’s Information Criterion, corrected for small samples (AIC_c_) [44]. All candidate models within ∼2 AIC_c_ units from the best-supported model (lowest AIC_c_ value) were considered to have reasonable support, given the data and set of models [44]. Relative support for each model was evaluated based on its Akaike weight, *w_i_*, ranging from zero (no support) to one (full support, relative to the other models considered) [44].

#### Wolf-pup survival

Year (1995–2007) and location effects on wolf-pup survival were evaluated using a logistic, generalized, linear, mixed model with random pack effects and AIC_c_ model-selection procedures. Not all monitored dens were visible from the air or ground, so we did not always have pup counts to match the serological results from a particular pack to directly test the relationship between seroprevalence and survival. Therefore, while the strength of inference was reduced, we used regression analyses to examine the relationship between annual wolf-pup survival and annual wolf-pup seroprevalence (*n* = 11 years of estimates), broken down by location (i.e., NR and Interior).

## Results

### Temporal, spatial, and demographic patterns of exposure

Wolf CPV seroprevalence was 100% across all years, locations, pups, and adults (Table 3; thus none of the wolf models were relevant for Table 4 or S2). The best-supported models (Table 4, Table S2) of coyote CPV seroprevalence also suggested either a constant probability of exposure of 0.94 for both adults (95% CI: 0.85, 0.98) and juveniles (95% CI: 0.79, 0.98) or a non-significant effect of resident status. Among juvenile coyotes, residents had a smaller probability of exposure (Pr[E]  = 0.92; 95% CI: 0.74, 0.98) than transients (Pr[E]  = 1; 95% CI: 0, 1), whereas among adults, the converse was true (residents: Pr[E]  = 0.98; 95% CI: 0.86, 1; transients: Pr[E]  = 0.86; 95% CI: 0.64, 0.95).

**Table 3:** Pathogen seroprevalence among canids in Yellowstone National Park, 1997–2007.

Pathogen	Category*	Wolf Seroprevalence	Coyote Seroprevalence
**CPV**	Pup/Juvenile	100% (117/117)	R: 92% (24/26)
			T: 100% (9/9)
	Adult	100% (92/92)	R: 98% (45/46)
			T: 86% (18/21)
**CAV-1**	Pup/Juvenile	91% (106/116)	R: 23% (6/26)
			T: 11% (1/9)
	Adult	96% (89/93)	R: 89% (41/46)
			T: 71% (15/21)
**CHV**	Total Population	87% (181/209)	R: 51% (39/77)
			T: 40% (12/30)
	Juvenile	84% (137/164)	23% (8/35)
	Young Adult	100% (39/39)	52% (28/54)
	Old Adult	83% (5/6)	87% (13/15)

* See Table 1 for a description of age categories.

Seroprevalence reported for canine parvovirus (CPV), canine adenovirus type-1 (CAV-1), and canine herpesvirus (CHV). Coyote seroprevalence is divided into residents (R) and transients (T). The fraction of (positives/total samples) are noted parenthetically. CHV analysis included age class as a risk factor, so analyses were not divided by pups/juveniles and adults.

**Table 4:** Top models of disease seroprevalence and survival for Yellowstone National Park’s canids.

Pathogen or Survival	Species & Age	Best-Supported Models	K	*n*	-Log Likeli-hood	AIC_c_	Δ	*w*
**Canine Parvovirus (CPV)**	**Coyote Juveniles**	CPV∼1	1	35	7.67	19.46	0.00	0.62
		CPV∼1+Resident	2	35	7.05	20.48	1.01	0.38
	**Coyote Adults**	CPV∼1+Resident	2	67	13.43	32.86	0.00	0.69
		CPV∼1	1	68	15.21	34.49	1.63	0.31
**Canine Adenovirus Type-1 (CAV)**	**Wolf Pups**	CAV∼1	1	116	14.34	32.73	0.00	0.74
		CAV∼1+Location	2	116	14.34	34.79	2.06	0.26
	**Wolf Adults**	CAV∼1	1	93	4.82	13.67	0.00	0.74
		CAV∼1+Location	2	93	4.82	15.76	2.09	0.26
	**Coyote Juveniles**	CAV∼1	1	35	17.47	39.06	0.00	0.67
		CAV∼1+Resident	2	35	17.05	40.47	1.40	0.33
	**Coyote Adults**	CAV∼1+Resident	2	67	28.37	62.93	0.00	0.88
**Canine Herpesvirus (CHV)**	**Wolves**	CHV∼1+AgeClass	3	209	56.97	122.02	0.00	0.98
	**Coyotes**	CHV∼1+AgeClass	3	104	61.52	131.24	0.00	0.46
		CHV∼1+Resident+AgeClass	4	103	60.43	131.31	0.07	0.45
***Neospora caninum (Neo)***	**Wolves**	Neo∼1+AgeClass+Year	13	202	53.10	136.14	0.00	0.28
		Neo∼1+AgeClass	3	202	64.17	136.42	0.29	0.24
		Neo∼1+Location+AgeClass	4	202	63.58	137.40	1.27	0.15
		Neo∼1+Year+Location+ AgeClass	14	202	52.73	137.75	1.61	0.12
**Canine Distemper Virus (CDV)**	**Wolf Pups**	CDV ∼1	1	114	42.46	88.97	0.00	0.65
		CDV ∼1+Year+Location	12	114	30.96	91.01	2.04	0.24
	**Wolf Adults**	CDV ∼1+Year	11	97	42.68	112.51	0.00	0.74
		CDV ∼1+Year+Location	12	97	42.45	114.61	2.11	0.26
	**Coyote Juveniles**	CDV ∼1	1	35	4.743	13.61	0.00	1.00
	**Coyote Adults**	CDV ∼1+Year	9	69	27.36	77.77	0.00	0.67
		CDV ∼1+Year+Resident	10	68	26.88	79.63	1.86	0.27
**Survival**	**Wolf Pups**	Survival∼1+Year+Location	14	723	363.10	756.79	0.00	0.82
		Survival∼1+Year+Location+ Location*Year	27	723	351.83	759.78	2.98	0.18

Models presented are those best-supported (Δ AIC_c_ <3) under the Information-theoretic approach [44]. Response variables include seroprevalence of canine parvovirus (CPV), canine adenovirus (CAV-1), canine herpesvirus (CHV), *Neospora caninum* (Neo), and canine distemper virus (CDV), as well as wolf-pup survival (Survival). Covariates are detailed in Table 1, but include Year, Location (Northern Range versus Interior; wolves only), Resident (resident versus transient status; coyotes only), and AgeClass (juvenile, young adult, or old adult). (K  =  number of estimable parameters, increasing differences from the best model (Δ) indicate decreasing model adequacy, and Akaike weights (*w*) express model support relative to all other models in the set. Additive effects are expressed with a plus sign, and interactions between factors are connected with an asterisk.).

The best-supported models for wolf-pup and adult CAV-1 seroprevalence suggested a constant, very high probability of exposure (for both pups and adults: Pr[E]  = 1, 95% CI: 0, 1), irrespective of year or location. Similar to CPV, the best-supported models for both juvenile and adult coyote CAV-1 exposure included a covariate for resident status. Although not significant, both juvenile and adult resident coyotes had greater probabilities of CAV-1 exposure (juveniles: Pr[E]  = 0.19; 95% CI: 0.02, 0.70; adults: Pr[E]  = 0.89, 95% CI: 0.76, 0.96) than their transient counterparts (juveniles: Pr[E]  = 0.07, 95% CI: 0.03, 0.18; adults: Pr[E]  = 0.72, 95% CI: 0.49, 0.87).

**Figure 1:**
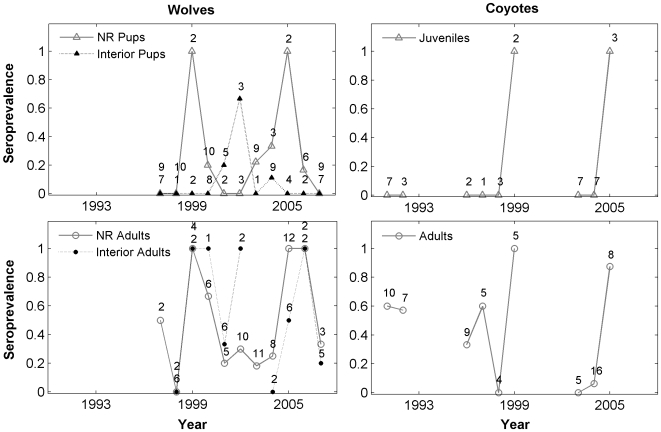
Annual canine distemper virus seroprevalence among wolves and coyotes in Yellowstone National Park, 1991–2007. Among wolves, data are divided by location (Northern Range [NR] and Interior), whereas coyotes were sampled only on the NR. Sample sizes are displayed above seroprevalences (see Table S3 for number of packs sampled and 95% CIs). Where points overlap, the top number refers to NR, the bottom to Interior.

By contrast, wolf and coyote exposure to CDV varied annually. The best-supported models for CDV exposure suggested constant, low pup exposure and a year effect among adults. There was also marginal support for a model with year and location effects among both wolf pups (Δ AIC_c_ = 2.04, weight = 0.24) and adults (Δ AIC_c_ = 2.11, weight = 0.26) which exhibited a much better fit, particularly to the pup data. Among adult coyotes, the best-supported models included year and resident effects. While the best-supported model for juvenile coyote seroprevalence suggested constant, near-zero exposure, the model exhibited poor fit to 2 years of the data (i.e., 1999 and 2005; Figure 1).

The probability of CDV exposure among wolf pups was highest in 1999, 2002, and 2005, a pattern less clearly mirrored in the adult data (no year effect was significant) (Figure 1, Table S3). Between these three outbreak years, there was evidence for a small amount of seroconversion among pups (20–33% in 2000, 2001, 2003, and 2004; Figure 1). In addition, both NR pups and adults had as much as a 36% and 14% positive difference, respectively, in their probability of exposure compared to their Interior counterparts (pups: OR = 4.25, 95% CI: 0.97, 18.54; adults: OR = 1.72; 95% CI: 0.36, 8.25).

Both juvenile and adult coyote seroprevalence mirrored the temporal patterns among NR wolf pups; CDV seroprevalence was 100% in 1999 and 2005 among both age groups and 0% otherwise among juveniles (year effects were not significant; Figure 1). Furthermore, adult resident coyotes had as much as an 18% positive difference in the probability of CDV exposure compared to adult transients (OR = 2.05, 95% CI: 0.41, 10.18), although this difference was not statistically significant.

The best-supported model for wolf exposure to CHV included a covariate for age class; however, wolf CHV seroprevalence was uniformly high (87%) and estimated probabilities of exposure were 1.0 for all three age classes (95% CIs, juveniles: 0.97–1.0; young adults: 0–1.0; old adults: 0–1.0). Among coyotes, the two competing models with nearly equal AIC_c_ weights suggested support for age class and resident status covariates in the risk of CHV exposure. The probability of CHV exposure among coyotes significantly increased with age class; juveniles had the lowest probability of exposure (Pr[E]  = 0.20, 95% CI: 0.09, 0.38; seroprevalence = 23%), followed by young adults (Pr[E]  = 0.81, 95% CI: 0.69, 0.89; seroprevalence = 51%), and old adults (Pr[E]  = 0.96, 95% CI: 0.83, 0.99; seroprevalence = 87%). Although not statistically significant, resident coyotes had as much as an 11% positive difference in their probability of CHV exposure compared to transients (OR = 1.58, CI: 0.59, 4.18).

**Figure 2:**
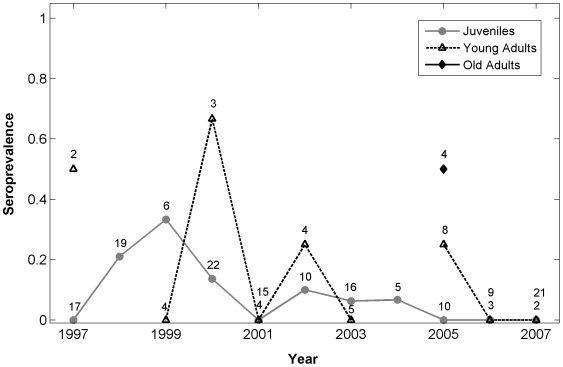
*Neospora caninum* seroprevalence among wolves in Yellowstone National Park, 1997–2007. Data are divided by age class: juvenile (0.5–1.9 yrs), young adult (2–4.9 yrs), and old adult (≥5 yrs). Sample sizes are displayed above seroprevalences. Where points overlap, numbers refer to juveniles, young adults and old adults, respectively.

The four best-supported models for *N. caninum* exposure among wolves suggested that age class, year, and location were important covariates (Table 4). Wolves’ probability of exposure increased with age; old adults had the greatest probability of exposure to *N. caninum* (Pr[E]  = 0.33, 95% CI: 0.08, 0.73), followed by young adults (Pr[E]  = 0.11, 95% CI: 0.10, 0.36), and juveniles (Pr[E]  = 0.04, 95% CI: 0.04, 0.13). There was no significant difference in year effects on the probability of exposure, although exposure in 2001, 2006, and 2007 was very low compared to other years (Figure 2). Furthermore, NR wolves had non-significant, greater probability of exposure (as much as a 14% difference) compared to Interior wolves (OR = 1.75, CI: 0.62, 4.94).

There were too few fox samples to look for patterns of exposure, but we did find evidence for fox exposure to CPV, CAV-1, CDV, and CHV (Table 5).

**Table 5:** Summary of red fox serological results, Yellowstone National Park, 1993–2005.

Year	*n*	CPV§	CAV-1*	CDV†	CHV‡
1993	3	2	0	0	0
1996	1	0	1	1	0
2003	3	2	3	0	1
2005	2	0	0	2	0

§ Canine parvovirus.

* Canine adenovirus type-1.

† Canine distemper virus.

‡ Canine herpesvirus.

Small samples (*n*) precluded analysis so number of positive cases are reported for each test instead.

### Wolf-pup survival and correlations with pathogen exposure

**Figure 3:**
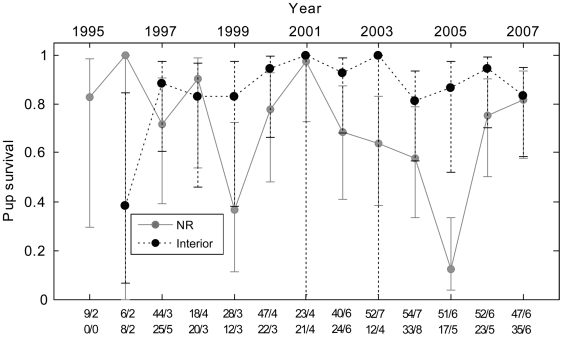
Annual wolf-pup survival in Yellowstone National Park by location (Northern Range [NR] and Interior), 1995–2007. Error bars are 95% confidence intervals and the numbers at the bottom of the graph represent the number of pups monitored/the number of packs observed (NR listed on top).

Between 1995 and 2007, we annually monitored an average of 9 (SD = 4, range = 2–15) wolf dens, or an average of 84% (SD = 14%) of reproducing packs. Although the best-supported model for annual wolf-pup survival included only year and location covariates, there was also model support for a year*location interaction (Table 4). Pup survival was significantly lower on the NR than in the Interior (OR = 0.25, 95% CI: 0.12, 0.49) (Figure 3). Although there was no significant year effect common to both NR and Interior wolves, the probability of survival was significantly lower among NR pups in 2005 (Pr[Survival]  = 0.13, 95% CI: 0.04, 0.33) (survival = 13%) when compared to most years, and lower than average, but not significantly so, in 1999 (Pr[Survival]  = 0.37, CI: 0.12, 0.71) (survival = 37%) (See Figure 3 for a comparison of 95% CIs). Although the 95% CIs on the NR survival estimates from 2005 overlap with those of 1995 and 1996, the latter’s confidence intervals are almost certainly too large. Survival estimates in these two years were derived from censuses of the small, closely monitored, reintroduced population, and thus were both accurate and precise.

Annual wolf-pup CDV seroprevalence coincided with significant variation in annual pup survival on the NR (*r* ^2^ = 0.69, *t* = −4.51, df = 10, *P* = 0.001), although this was not the case in the Interior (*r* ^2^ = 0.02, *t* = 0.48, df = 10, *P* = 0.65). None of the other viral pathogens (CPV, CAV-1, and CHV) exhibited significant temporal variation capable of explaining temporal patterns of pup survival. Annual wolf-pup survival was independent of annual pup exposure to *N. caninum* (NR: *r* ^2^ = 0.18, *t* = −1.39, df = 10, *P* = 0.20; Interior: *r* ^2^ = 0.09, *t* = −0.91, df = 10, *P* = 0.38).

## Discussion

The discussion that follows must be qualified by the fact that overall, our sample sizes were quite small. Small samples reduced our accuracy and precision in estimating exposure rates as well as our power to detect significant differences in exposure between groups, hence limiting the strength of our inferences based on our data. This is particularly apparent in our analysis of CDV exposure, where our supported models included many estimable parameters. However, in some cases, small samples were unavoidable. For example, in 1999 and 2005, pup survival was so poor that only 13 and 8 pups, respectively, were known to be alive on the NR, making it very difficult to capture pups in those years.

Our conclusions must further be qualified by the fact that our serological assays were not specifically validated or optimized for wolves, coyotes, or foxes. Without knowing the sensitivity and specificity of our tests for these species, we do not know the degree to which our positive and negative test results reflect true exposure status. We cannot rule out, for example, false positive results caused by non-specific antibody binding or exposure to closely related or cross-reacting viruses. However, there is good biological reason to believe that wolf and coyote immune systems would behave very similarly to those of closely related domestic dogs, for which the tests have been optimized. Previous serological work with foxes (including CDV assay validation via vaccination trials) suggests our titer cutoffs were appropriate for this species as well [32]. Furthermore, the fact that multiple species exhibited similar patterns of exposure suggests that we did detect ‘real’ signals of disease exposure.

Our findings suggest CPV, CAV-1, and CHV are enzootic, and that CDV is epizootic, within Yellowstone’s canid community. Among wolves, *N. caninum* appears enzootic, although it does exhibit some temporal variation, possibly reflecting complex dynamics between the parasite, its intermediate hosts (domestic and wild ungulates), and definitive hosts (wild canids and domestic dogs).

Resident status of coyotes was the one variable that consistently emerged as a possible risk factor, regardless of age group or pathogen. Contrary to our original hypothesis, resident coyotes tended to have a greater probability of pathogen exposure than their transient counterparts. We had hypothesized that transient coyotes, whose home-ranges overlap multiple resident packs’ territories, might contact a greater variety of individuals and be at greater risk for pathogen exposure. However, it is possible that transients actually make fewer contacts with other coyotes compared to residents, whose frequent interactions among pack-mates may provide the best opportunity for pathogen transmission. An alternative explanation, at least among adult coyotes (≥1.6 yrs), is that the sampled residents tended to be slightly older (10% of transient adult coyotes were old adults [i.e., the other 90% were young adults] compared to the 28% of resident adult coyotes that were old adults), and that perhaps age, which should be positively correlated with exposure risk, was a confounder. From our study alone, it is not clear whether social status among coyotes has a true effect on the pathogen-exposure risk as hypothesized for other social-mammal systems [45], [46].

### Canine parvovirus

Following the emergence of CPV in the late 1970s, studies throughout North America have reported high seroprevalences for CPV among wild canids [47]–[49]. Nearly all wolves and coyotes that we tested in YNP were positive for CPV exposure by 0.5–0.75 yrs of age. This high seroprevalence suggests low levels of disease-induced host mortality [50], [51] and high rates of transmission, perhaps aided by the stability of CPV in the environment [52]. We did not detect evidence for CPV-induced wolf-pup mortality, contrary to reports of suspected or confirmed CPV-induced mortality in the 1980s and early 1990s [53]–[55], including among coyote pups in YNP [47]. Furthermore, CPV seroprevalence offered no explanation for pup-survival patterns because there was no annual variation in exposure to CPV among wolves or coyotes. However, it is possible that CPV either causes a constant, low level of mortality or periodic mortality when combined with other factors such as nutritional stress or co-infection with other pathogens, both scenarios of which our current methods would fail to detect.

### Canine adenovirus type-1

Nearly all wolves also exhibited exposure to CAV-1, but there was no evidence for or against disease-induced mortality. CAV-1 seroprevalence has generally been high in other canid surveys [47], [56]–[58], suggesting that transmission among wild canids is high. Juvenile coyotes had much lower seroprevalences to CAV-1 than did wolf pups, but this may have been due, in part, to the slightly younger age at which coyotes were sampled.

### Canine herpesvirus

None of the studies that screened for CHV antibodies among wild canids found evidence for exposure ([59] (*Canis lupus*); [56] (*Canis latrans*); [60] (*Chrysocyon brachyurus*)). By contrast, CHV seroprevalence was high among YNP wolves, but somewhat lower and age-dependent in coyotes. Canine herpesvirus is primarily spread though direct contact, so wolves’ higher seroprevalence may be attributed to higher contact rates or a greater variety of contacts compared to coyotes or foxes. Similarly, relatively high intra-pack contact rates may help explain the trend towards a slightly higher risk of pathogen exposure among resident coyotes compared to their transient counterparts. Furthermore, increasing risk of exposure with age, as observed among wolves and coyotes, is common for enzootic diseases.

#### Neospora caninum

*N. caninum* exposure among wolves suggests that a sylvatic cycle of this protozoan parasite exists in YNP. Domestic livestock, except horses, are prohibited in YNP. As hoofed-stock-to-canid transmission occurs through ingestion of infected tissue, livestock is likely not the source of canid exposure to *N. caninum* in YNP. While there is no information on *N. caninum* prevalence among YNP ungulates, other studies have detected *N. caninum* antibodies among deer (*Odocoileus virginianus*) [61], [62], bison, and moose (*Alces alces*) [63]. Thus, wild ungulates are suspected to be intermediate hosts and the source of exposure for YNP wolves. While wolves have not been shown to shed *N. caninum* oocysts, given the wolf’s genetic similarity to dogs and coyotes, both of which shed oocysts [20], [64], infected wolves probably shed them as well. Although we had insufficient quantities of coyote and fox sera to screen for *N. caninum*, we suspect that exposure levels in at least coyotes would be similar to that of YNP wolves. We did not sample wild canids outside of YNP, and thus future research could employ a combination of serologic and genetic tools to look at the relationships between *N. caninum* in wild and domestic canids and ungulates in regions where *N. caninum* is of concern to local livestock producers. However, at this time, there is no evidence to suggest that *N. caninum* has been or will be significantly impacting either domestic or wild ungulates or canids in the GYE.

### Canine distemper virus

The dynamics of highly immunizing, fast acting, epidemic-type pathogens such as CDV are challenging to decipher within the usual 3–5-year time frame of most wildlife studies. In these situations, reports of average seroprevalence, without regard to year or age of the sampled animal, can be misleading and of limited value for comparisons across different study sites and populations. In many of the serosurveys among coyotes [47], [65]–[67] and wolves [55], [68], dynamic temporal patterns may have been masked by examining CDV seroprevalence averaged across years or age classes at spatial scales likely too small for CDV to be enzootic [69].

The supported CDV seroprevalence models suggested that (1) coyotes experienced CDV outbreaks in 1999 and 2005, (2) all wolves experienced CDV outbreaks in 1999, 2002, and 2005 (although 2000 and 2006 adult wolf seroprevalence was also high, these were likely individuals that were exposed in 1999 and 2005 and were thus positive upon capture the following year), and (3) NR wolves experienced a greater probability of CDV exposure than Interior wolves. This last finding was consistent our hypothesis that high wolf densities on the NR may result in higher inter-pack contact rates and thus higher levels of pathogen exposure compared to the less-dense Interior. Although we do not have Interior density estimates for the other canids, it is quite possible that coyote and fox densities are also higher on the NR than in the Interior, and thus higher canid densities in general may contribute to higher rates of wolf exposure observed on the NR.

However, as the seroprevalence data suggested, these aforementioned generalities obscured some potentially important differences in spatial and temporal CDV dynamics. For example, none of the Interior wolf pups handled in 1999 and 2005 had been exposed to CDV in contrast to the high levels of exposure found among the limited samples of Interior adults and NR adults and pups. These inconsistencies may be the result of small samples or differences in case-fatality rates across sampling locations. If all infected pups in the Interior died due to disease, those available for sampling would all be negative. It is also possible that the timing and point of disease introduction into YNP could account for these differences. CDV is generally thought to move quickly through populations as it is highly contagious, infected individuals shed virus for a relatively short time (mean duration of infectiousness  = 14 days, maximum 90 days), and the virus rapidly degrades in the environment (within hours at ≥20°C, and within several weeks at 0–4°C) [15], [16]. Thus, if CDV had entered from the south before pup birth or weaning, it could have swept through the Interior adults, sparing the young Interior pups protected by maternal antibodies but arriving on the NR when pups would be most vulnerable.

Furthermore, if there was in fact a 2002 outbreak, it seems to have been confined to the Interior wolves; none of the NR pups and only a few of the NR adults were exposed that year. However, there may be reason to suspect false positives in this particular case. In 2002, the two positive Interior pups had antibody titers just over the positive titer cutoff value (Positive antibody titer: ≥16), in contrast to marked increases in the titers observed among NR pups in 1999 and 2005 (Figure S1). Adult titers in the Interior and NR were not particularly high in 2002, either. In the absence of larger samples and more conclusive evidence (e.g. virus isolation or identification via PCR), we cannot be sure that CDV actually swept through the Interior of the park in 2002.

The wolf-pup data suggested low rates of seroconversion between the discrete outbreak years of 1999, 2002, and 2005. Once a wild or domestic canid is infected with CDV, the animal either recovers rapidly (mean time from infection to recovery [including latency and infectiousness]  = 21 days, maximum 120 days) with life-long immunity or dies [15], [16]. Thus, CDV requires a large population of susceptibles to persist, a population likely larger than YNP’s canid community [69]. The low seroconversion between epizootics, if representative of true positives, suggests re-exposure from some wild or domestic host outside YNP or mistaken assumptions about the disease. For example, although no evidence exists for carrier states, loss of immunity, or imperfect protection against novel strains of CDV among canids, loss of CDV immunity has been documented in raccoons (*Procyon lotor*) [70]. If any of these factors pertained to canids, that could help explain the apparent persistence of CDV in YNP. Perhaps more likely, as there are multiple competent hosts for CDV within the GYE (e.g. short and long-tailed weasels, American martens, striped skunks, and raccoons), multi-host transmission might allow localized CDV persistence within the GYE.

### Canine distemper virus exposure and wolf-pup survival

Although a thorough analysis of factors influencing wolf-pup survival would evaluate multiple hypotheses such as population density and food availability, the strong negative correlation between NR CDV seroprevalence and NR wolf-pup survival supports the hypothesis that CDV may have contributed to high NR pup mortality in 1999 and 2005. Although ≥8 young wolf-pup carcasses were located in 2005, all were too degraded for CDV isolation. We found several pup mandibles (*n* = 4) and handled two live pups during the winter of 2005–2006 displaying the distinctive tooth-enamel hypoplasia diagnostic of CDV [12], [71], [72]. Furthermore, several coyote dens appeared to experience high pup loss in 2005, with pups displaying neurologic symptoms consistent with late-stage CDV infection (E. Almberg, personal observation) [16]. More recent data suggests that CDV swept through the park again in 2008, and in addition to observing the same patterns of high CDV seroprevalence and very low wolf-pup survival, we recovered CDV ribonucleic acid via PCR from 3 dead wolves, all of which had been born after 2005 and thus presumably had no acquired immunity against CDV (Almberg, unpublished data). Despite the negative correlation between CDV exposure and pup survival, however, the ultimate causes of death could have been due to synergistic effects of CDV and another pathogen (e.g. CPV, CAV-1, canine coronavirus, or protozoan or helminth infections), such as with CDV and *Babesia* in Serengeti’s lions [73]. Population impacts of pup mortality were short term, for the wolf population rebounded in both years following the 1999 and 2005 lows [27].

We found no relationship between Interior CDV seroprevalence and Interior wolf-pup survival. Aside from the hypothesis that the timing of CDV introduction into the Interior either happened to be too early (e.g. 1999 and 2005) or too late (e.g. in 2002) to cause significant pup mortality, other plausible explanations for this lack of relationship include 1) that there was no CDV outbreak in 2002, and thus insufficient variation in exposure to detect a relationship with survival, and 2) that we failed to detect pup mortality due to bias in our sampling methods. The Interior packs’ dens were remote and only visible from the airplane, and thus, on average, we made our first pup observations and obtained our first high counts of pups over a month later than those made on the NR (First pup observations, NR: μ_date_ = 5/24, sd = 21 days, Interior: μ_date_ =  6/26, sd = 27 days; First high pup count, NR: μ_date_ = 6/19, sd = 34 days, Interior: μ_date_ = 7/22, sd = 37 days). Because much microparasite-induced (e.g., viruses and bacteria) pup mortality takes place following weaning (i.e., at 10–12 weeks of age) in late June through early July, it is quite possible that we failed to detect most Interior pup mortality, yielding artificially high survival estimates.

### Evidence for multi-host CDV outbreaks

The results of two previous studies on pathogen exposure in YNP carnivores further support the patterns of CDV exposure that we observed in wolves and coyotes. Gese *et al.* [47] suggested that YNP coyotes experienced a CDV outbreak between 1989 and 1991, which fits with the ∼50% seroprevalence we detected in adult coyotes sampled during 1991. Also, cougars in YNP appeared to experience isolated outbreaks of CDV in 1991 and 1999 [74], lending support to the pattern of discrete, multi-host, CDV epizootics. Our own extremely limited fox data at least did not contradict the pattern of discrete CDV outbreaks; the single positive animal sampled in 1996 was ≥5 years old and thus could have been exposed as a kit during the 1989/90 outbreak, and the only other two positive animals were sampled in 2005. Furthermore, mustelids are highly susceptible to CDV, and the badger population on the NR appeared to have crashed in 2005 (E. Almberg, personal observation). However, there are no data on CDV exposure or survival patterns among mustelids in YNP.

These correlations among multiple hosts suggest regular CDV spillover but might also suggest multi-host transmission contributing to CDV persistence in the larger region. Domestic animals cannot be ruled out as a reservoir for CDV. However, reported CDV cases in Montana’s domestic animals are uncommon, with 18 possible cases recorded between 1994 and 2008 (Montana Veterinary Diagnostic Lab, Bozeman, MT, USA, unpublished data). Furthermore, while the percentage of local domestic animals vaccinated for CDV is unknown, it is probably safe to assume that the unvaccinated population of dogs and cats is too small to serve as a CDV reservoir [69].

YNP and the GYE are not closed biological systems. On an annual basis, an unknown number of visitors from around the U.S. bring their pets to YNP and the GYE. There is currently no proof of dog health or immunization required for entry into the national parks. Visiting domestic animals certainly constitute a plausible route for new or emerging pathogens (particularly those that are vector-borne or indirectly transmitted) to enter into local, wild canid populations. Furthermore, YNP is a small fraction of the overall GYE, and pathogen dynamics within YNP may be in part a product of much larger-scale dynamics driven by inter-connected canid and carnivore populations in the Rocky Mountains.

In summary, the constant high canid exposure to CPV, CAV-1, and CHV in YNP suggest that these pathogens are established in the wolf and coyote populations and that they are unlikely to be causing acute mortality in their hosts [50], [51]. Although *N. caninum* is unlikely to impact canid health, wolf exposure indicates a sylvatic cycle in the park, which may or may not be related to the parasite’s dynamics among regional livestock. Canine distemper appears to cycle through YNP’s carnivores in periodic epizootics, and may have contributed to low wolf-pup survival in 1999 and 2005 on the NR. Although CDV does not appear to jeopardize the long-term population survival of YNP wolves, it can cause short-term population decreases. Additional information on how and where CDV is maintained and the frequency with which future epizootics might be expected would be useful for regional managers working on canids in the Northern Rocky Mountains.

## Supporting Information

Table S1 Epidemiological characteristics of selected canid pathogens. Data are largely based on the study of domestic dogs.
(0.04 MB DOC)

Table S2 Models of disease seroprevalence and survival considered and evaluated for Yellowstone National Park's canids. Response variables include seroprevalence of canine parvovirus (CPV), canine adenovirus (CAV-1), canine herpesvirus (CHV), Neospora caninum (Neo), and canine distemper virus (CDV), as well as wolf-pup survival (Survival). Covariates are detailed in Table 1, but include Year, Location (Northern Range versus Interior; wolves only), Resident (resident versus transient status; coyotes only), and AgeClass (juvenile, young adult, or old adult). (K  =  number of estimable parameters, increasing differences from the best model (□) indicate decreasing model adequacy, and Akaike weights (w) express model support relative to all other models in the set. Additive effects are expressed with a plus sign, and interactions between factors are connected with an asterisk.)
(0.14 MB DOC)

Table S3 Wolf and coyote canine distemper seroprevalence and associated 95% score confidence intervals. Sample sizes and the number of packs (for wolves) or regions (for coyotes) sampled are noted.
(0.14 MB DOC)

Figure S1 Mean wolf antibody titers to canine distemper virus in Yellowstone National Park, 1997–2007. Mean log_2_(antibody titers) are displayed with corresponding 95% confidence intervals for Northern Range (NR) and Interior pups (A) and adults (B).
(7.71 MB TIF)
