# Supplementary material for: A Serological Survey of Infectious Disease in Yellowstone National Park’s Canid Community
Source: PLoS One. 2009 Sep 16;4(9):e7042. doi: 10.1371/journal.pone.0007042 (PMC2738425; doi:10.1371/journal.pone.0007042)
Supplement: Table S1 — Epidemiological characteristics of selected canid pathogens. Data are largely based on the study of domestic dogs. (0.04 MB DOC) [file pone.0007042.s001.doc]

**Table S1. Epidemiological characteristics of selected canid pathogens.**

| **Pathogen** | **Transmission** | **Duration of infectiousness** | **Infectious period outside host** | **Symptoms** | **Course of infection** | **Mortality pattern in domestic carnivores** | **References** |
| --- | --- | --- | --- | --- | --- | --- | --- |
| Canine parvovirus (CPV) | Direct contact with oral and nasal exudates, and indirect fecal-oral contact | Shedding begins 4 days post-infection (PI) and generally ceases by 30 days PI | Extremely stable in the environment; 6 months at 20 º C | Lympho-penia, anemia, vomiting, diarrhea, and dehydration | Mild to acute gastroenter-itis, followed by clearance or occasional carrier status | In un-vaccinated populations, mortality is greatest in  pups <1 yr | [14 ] |
| Canine distemper virus (CDV) | Direct contact with respiratory exudates (aerosol) | Shedding begins 7 days PI and either lasts through day 14 PI and is cleared, the animal dies, or the infection becomes subclinical and persists for 60-90 days | 1-3 h at 37º C, weeks at < 4º C | Lympho-penia, fever, nasal and conjunctival discharges, anorexia, vomiting, diarrhea, myoclonus, encephalitis, immuno-suppression | Acute infection is followed by complete clearance or subacute/ persistent infection in CNS | In un-vaccinated populations, mortality is greatest in  pups <1 yr | [15] |
| Canine adenovirus type-1 (CAV-1) | Direct contact with nasal and conjunctival secretions, urine, or feces, or indirect through contact with contaminated fomites | Viremia lasts 4-8 days PI, but shedding in urine can persist for 6-9 months | Days at 20º C, months at < 4º C | Leukopenia, fever, apathy, anorexia, vomiting, and diarrhea. May develop broncho-pneumonia, conjuncti-vitis, photophobia and transient corneal opacity (“blue eye”) | Virus is either quickly cleared or causes acute/chronic hepatitis. Following full recovery, immunity is likely lifelong | In un-vaccinated populations, mortality is greatest in  pups <1 yr | [17,18] |
| Canine herpesvirus (CHV) | Direct contact with oral, nasal, and genital secretions; transplacental | Lifelong infection with shedding variable and reactivated by stress, immuno-suppression, and/or pregnancy | 22 hrs at 37º C | *Adults*: Mild upper respiratory infection; genital lesions; abortion  *Neonates*: Lethargy, anorexia, weight loss, rhinitis, and rash | Following initial clinical/sub-clinical infections, latent infection persists for months-to-years and is intermittently reactivated | Fetal and neonate mortality are greatest | [19] |
| *Neospora caninum* (protozoan) | Canids consuming infected wild or domestic ungulate tissues; transplacental | Shedding of oocysts starts 5 days PI and can last for several months; Infection is lifelong and shedding can either be chronic or reactivated during periods of stress or pregnancy | Unknown | Most infections are likely subclinical and asympto-matic  *Acute disease:* neurological and muscular disorder (paralysis in pups), hepatic, pulmonary, and myocardial dysfunction, fever and vomiting | Following initial clinical/sub-clinical infection, infection is either chronic or subclinical and can be reactivated during periods of stress or pregnancy | While mortality is generally uncommon, pups are most susceptible | [21] |

Data are largely based on the study of domestic dogs.
