# Supplementary material for: A Serological Survey of Infectious Disease in Yellowstone National Park’s Canid Community
Source: PLoS One. 2009 Sep 16;4(9):e7042. doi: 10.1371/journal.pone.0007042 (PMC2738425; doi:10.1371/journal.pone.0007042)
Supplement: Table S2 — Models of disease seroprevalence and survival considered and evaluated for Yellowstone National Park's canids. Response variables include seroprevalence of canine parvovirus (CPV), canine adenovirus (CAV-1), canine herpesvirus (CHV), Neospora caninum (Neo), and canine distemper virus (CDV), as well as wolf-pup survival (Survival). Covariates are detailed in Table 1, but include Year, Location (Northern Range versus Interior; wolves only), Resident (resident versus transient status; coyotes only), and AgeClass (juvenile, young adult, or old adult). (K = number of estimable parameters, increasing differences from the best model (□) indicate decreasing model adequacy, and Akaike weights (w) express model support relative to all other models in the set. Additive effects are expressed with a plus sign, and interactions between factors are connected with an asterisk.) (0.14 MB DOC) [file pone.0007042.s002.doc]

**Table S2.** **Models of disease seroprevalence and survival considered and evaluated for Yellowstone National Park’s canids.**

| **Pathogen or Survival** | **Species & Age** | **Model** | **K** | ***n*** | **-Log Likeli-hood** | **AIC_c_** | **Δ** | ***w*** |
| --- | --- | --- | --- | --- | --- | --- | --- | --- |
| **Canine Parvovirus (CPV)** | **Coyote Juveniles** | CPV~1 | 1 | 35 | 7.67 | 19.46 | 0.00 | 0.62 |
|  |  | CPV~1+Resident | 2 | 35 | 7.05 | 20.48 | 1.01 | 0.38 |
|  |  | CPV~1+Year | 9 | 35 | 4.78 | 36.76 | 17.30 | 0.00 |
|  |  | CPV~1+Year+Resident | 10 | 35 | 3.64 | 38.44 | 18.98 | 0.00 |
|  | **Coyote Adults** | CPV~1+Resident | 2 | 67 | 13.43 | 32.86 | 0.00 | 0.69 |
|  |  | CPV~1 | 1 | 68 | 15.21 | 34.49 | 1.63 | 0.31 |
|  |  | CPV~1+Year | 9 | 68 | 10.53 | 44.15 | 11.30 | 0.00 |
|  |  | CPV~1+Year+Resident | 10 | 67 | 9.26 | 44.45 | 11.59 | 0.00 |
| **Canine Adenovirus Type-1 (CAV)** | **Wolf Pups** | CAV~1 | 1 | 116 | 14.34 | 32.73 | 0.00 | 0.74 |
|  |  | CAV~1+Location | 2 | 116 | 14.34 | 34.79 | 2.06 | 0.26 |
|  |  | CAV~1+Year | 11 | 116 | 13.88 | 54.30 | 21.57 | 0.00 |
|  |  | CAV~1+Year+Location | 12 | 116 | 13.84 | 56.71 | 23.98 | 0.00 |
|  |  | CAV~1+Year+Location+ Year*Location | 22 | 116 | 12.57 | 82.01 | 49.29 | 0.00 |
|  | **Wolf Adults** | CAV~1 | 1 | 93 | 4.82 | 13.67 | 0.00 | 0.74 |
|  |  | CAV~1+Location | 2 | 93 | 4.82 | 15.76 | 2.09 | 0.26 |
|  |  | CAV~1+Year | 11 | 93 | 4.73 | 36.71 | 23.04 | 0.00 |
|  |  | CAV~1+Year+Location | 12 | 93 | 5.61 | 41.12 | 27.45 | 0.00 |
|  |  | CAV~1+Year+Location+ Year*Location | 22 | 93 | 4.55 | 69.55 | 55.87 | 0.00 |
|  | **Coyote Juveniles** | CAV~1 | 1 | 35 | 17.47 | 39.06 | 0.00 | 0.67 |
|  |  | CAV~1+Resident | 2 | 35 | 17.05 | 40.47 | 1.40 | 0.33 |
|  |  | CAV~1+Year | 9 | 35 | 13.75 | 54.70 | 15.64 | 0.00 |
|  |  | CAV~1+Year+Resident | 10 | 35 | 12.72 | 56.61 | 17.55 | 0.00 |
|  | **Coyote Adults** | CAV~1+Resident | 2 | 67 | 28.37 | 62.93 | 0.00 | 0.88 |
|  |  | CAV~1 | 1 | 68 | 31.65 | 67.35 | 4.42 | 0.10 |
|  |  | CAV~1+Year+Resident | 10 | 67 | 22.46 | 70.84 | 7.91 | 0.02 |
|  |  | CAV~1+Year | 9 | 68 | 24.39 | 71.88 | 8.96 | 0.01 |
| **Canine Herpesvirus (CHV)** | **Wolves** | CHV~1+AgeClass | 3 | 209 | 56.97 | 122.02 | 0.00 | 0.98 |
|  |  | CHV~1 | 1 | 209 | 63.28 | 130.62 | 8.60 | 0.01 |
|  |  | CHV~1+Location | 2 | 209 | 63.24 | 132.56 | 10.54 | 0.01 |
|  |  | CHV~1+Year+AgeClass | 13 | 209 | 51.37 | 134.57 | 12.55 | 0.00 |
|  |  | CHV~1+Location+Year+ AgeClass | 15 | 209 | 51.09 | 136.69 | 14.67 | 0.00 |
|  |  | CHV~1+Year | 11 | 209 | 57.01 | 141.34 | 19.32 | 0.00 |
|  |  | CHV~1+Year+Location | 12 | 209 | 56.70 | 142.99 | 20.97 | 0.00 |
|  |  | CHV~1+Location+Year+ Location*Year | 22 | 209 | 47.15 | 149.72 | 27.70 | 0.00 |
|  | **Coyotes** | CHV~1+AgeClass | 3 | 104 | 61.52 | 131.24 | 0.00 | 0.46 |
|  |  | CHV~1+Resident+AgeClass | 4 | 103 | 60.43 | 131.31 | 0.07 | 0.45 |
|  |  | CHV~1+Resident+AgeClass+Year | 12 | 103 | 52.48 | 134.47 | 3.23 | 0.09 |
|  |  | CHV~1+Resident | 2 | 107 | 72.37 | 150.82 | 19.58 | 0.00 |
|  |  | CHV~1 | 1 | 110 | 75.13 | 154.34 | 23.10 | 0.00 |
|  |  | CHV~1+Year | 9 | 110 | 69.51 | 160.80 | 29.56 | 0.00 |
| ***Neospora caninum (Neo)*** | **Wolves** | Neo~1+AgeClass+Year | 13 | 202 | 53.10 | 136.14 | 0.00 | 0.28 |
|  |  | Neo~1+AgeClass | 3 | 202 | 64.17 | 136.42 | 0.29 | 0.24 |
|  |  | Neo~1+Location+AgeClass | 4 | 202 | 63.58 | 137.40 | 1.27 | 0.15 |
|  |  | Neo~1+Year+Location+ AgeClass | 14 | 202 | 52.73 | 137.75 | 1.61 | 0.12 |
|  |  | Neo~1+Year | 11 | 202 | 56.69 | 138.79 | 2.65 | 0.07 |
|  |  | Neo~1 | 1 | 202 | 67.41 | 138.82 | 2.68 | 0.07 |
|  |  | Neo~1+Location | 2 | 202 | 66.98 | 140.06 | 3.92 | 0.04 |
|  |  | Neo~1+Year+Location | 12 | 202 | 56.36 | 140.35 | 4.21 | 0.03 |
| **Canine Distemper Virus (CDV)** | **Wolf Pups** | CDV~1 | 1 | 114 | 42.46 | 88.97 | 0.00 | 0.65 |
|  |  | CDV~1+Year+Location | 12 | 114 | 30.96 | 91.01 | 2.04 | 0.24 |
|  |  | CDV~1+Year | 11 | 114 | 32.99 | 92.56 | 3.59 | 0.11 |
|  |  | CDV~1+Year+Location+ Year*Location | 22 | 114 | 21.94 | 100.99 | 12.03 | 0 |
|  | **Wolf Adults** | CDV~1+Year | 11 | 97 | 42.68 | 112.51 | 0 | 0.74 |
|  |  | CDV~1+Year+Location | 12 | 97 | 42.45 | 114.61 | 2.11 | 0.26 |
|  |  | CDV~1 | 1 | 97 | 61.05 | 126.14 | 13.64 | 0 |
|  |  | CDV~1+Year+Location+ Year*Location | 22 | 97 | 35.92 | 129.52 | 17.01 | 0 |
|  | **Coyote Juveniles** | CDV~1 | 1 | 35 | 4.743 | 13.61 | 0 | 1 |
|  |  | CDV~1+Year | 9 | 35 | 4.34E-10 | 27.2 | 13.59 | 0 |
|  |  | CDV~1+Year+Resident | 10 | 35 | 4.34E-10 | 31.17 | 17.56 | 0 |
|  | **Coyote Adults** | CDV~1+Year | 9 | 69 | 27.36 | 77.77 | 0 | 0.67 |
|  |  | CDV~1+Year+Resident | 10 | 68 | 26.88 | 79.63 | 1.86 | 0.27 |
|  |  | CDV~1 | 1 | 69 | 39.22 | 82.51 | 4.74 | 0.06 |
| **Survival** | **Wolf Pups** | Survival~1+Year+Location | 14 | 723 | 363.1 | 756.79 | 0 | 0.82 |
|  |  | Survival~1+Year+Location+ Location*Year | 27 | 723 | 351.83 | 759.78 | 2.98 | 0.18 |
|  |  | Survival~1+Year | 13 | 723 | 371 | 770.51 | 13.72 | 0 |
|  |  | Survival~1 | 1 | 723 | 384.2 | 772.41 | 15.61 | 0 |

Response variables include seroprevalence of canine parvovirus (CPV), canine adenovirus (CAV-1), canine herpesvirus (CHV), *Neospora caninum* (Neo), and canine distemper virus (CDV), as well as wolf-pup survival (Survival). Covariates are detailed in Table 1, but include Year, Location (Northern Range versus Interior; wolves only), Resident (resident versus transient status; coyotes only), and AgeClass (juvenile, young adult, or old adult). (K = number of estimable parameters, increasing differences from the best model (∆) indicate decreasing model adequacy, and Akaike weights (*w*) express model support relative to all other models in the set. Additive effects are expressed with a plus sign, and interactions between factors are connected with an asterisk.)
