# Supplementary material for: A Serological Survey of Infectious Disease in Yellowstone National Park’s Canid Community
Source: PLoS One. 2009 Sep 16;4(9):e7042. doi: 10.1371/journal.pone.0007042 (PMC2738425; doi:10.1371/journal.pone.0007042)
Supplement: Table S3 — Wolf and coyote canine distemper seroprevalence and associated 95% score confidence intervals. Sample sizes and the number of packs (for wolves) or regions (for coyotes) sampled are noted. (0.14 MB DOC) [file pone.0007042.s003.doc]

**Table S3. Wolf and coyote canine distemper seroprevalence and associated 95% score confidence intervals.**

|  | **Year** | ***n*** | **No. of packs or sampling locations** | **Seroprevalence** | **95% CI** |
| --- | --- | --- | --- | --- | --- |
| **Wolves** |  |  |  |  |  |
| **NR Pups** | 1997 | 9 | 3 | 0 | (0, 0.30) |
|  | 1998 | 10 | 4 | 0 | (0, 0.28) |
|  | 1999 | 2 | 2 | 1 | (0.34, 1) |
|  | 2000 | 10 | 3 | 0.20 | (0.06, 0.51) |
|  | 2001 | 2 | 2 | 0 | (0, 0.66) |
|  | 2002 | 3 | 3 | 0 | (0, 0.56) |
|  | 2003 | 9 | 4 | 0.22 | (0.06, 0.55) |
|  | 2004 | 3 | 3 | 0.33 | (0.06, 0.79) |
|  | 2005 | 2 | 2 | 1 | (0.34, 1) |
|  | 2006 | 6 | 4 | 0.17 | (0.03, 0.56) |
|  | 2007 | 9 | 5 | 0 | (0, 0.30) |
| **Interior Pups** | 1997 | 7 | 4 | 0 | (0, 0.35) |
|  | 1998 | 1 | 1 | 0 | (0, 0.79) |
|  | 1999 | 2 | 2 | 0 | (0, 0.66) |
|  | 2000 | 8 | 3 | 0 | (0, 0.32) |
|  | 2001 | 5 | 3 | 0.20 | (0.04, 0.62) |
|  | 2002 | 3 | 2 | 0.67 | (0.21, 0.94) |
|  | 2003 | 1 | 1 | 0 | (0, 0.79) |
|  | 2004 | 9 | 4 | 0.11 | (0.02, 0.44) |
|  | 2005 | 4 | 2 | 0 | (0, 0.49) |
|  | 2006 | 2 | 1 | 0 | (0, 0.66) |
|  | 2007 | 7 | 5 | 0 | (0, 0.35) |
| **NR Adults** | 1997 | 2 | 2 | 0.50 | (0.09, 0.91) |
|  | 1998 | 2 | 2 | 0 | (0, 0.66) |
|  | 1999 | 4 | 3 | 1 | (0.51, 1) |
|  | 2000 | 6 | 4 | 0.67 | (0.30, 0.90) |
|  | 2001 | 5 | 2 | 0.20 | (0.04, 0.62) |
|  | 2002 | 10 | 7 | 0.30 | (0.11, 0.60) |
|  | 2003 | 11 | 4 | 0.18 | (0.05, 0.48) |
|  | 2004 | 8 | 4 | 0.25 | (0.07, 0.59) |
|  | 2005 | 12 | 5 | 1 | (0.76, 1) |
|  | 2006 | 2 | 2 | 1 | (0.34, 1) |
|  | 2007 | 3 | 3 | 0.33 | (0.06, 0.79) |
| **Interior Adults** | 1997 | 0 | 0 |  |  |
|  | 1998 | 6 | 3 | 0 | (0, 0.39) |
|  | 1999 | 2 | 1 | 1 | (0.34, 1) |
|  | 2000 | 1 | 1 | 1 | (0.21, 1) |
|  | 2001 | 6 | 3 | 0.33 | (0.10, 0.70) |
|  | 2002 | 2 | 1 | 1 | (0.34, 1) |
|  | 2003 | 0 | 0 |  |  |
|  | 2004 | 2 | 1 | 0 | (0, 0.66) |
|  | 2005 | 6 | 4 | 0.50 | (0.19, 0.81) |
|  | 2006 | 2 | 1 | 1 | (0.34, 1) |
|  | 2007 | 5 | 4 | 0.20 | (0.04, 0.62) |
| **Coyotes** |  |  |  |  |  |
| **NR Juveniles** | 1991 | 7 | 2 | 0 | (0, 0.35) |
|  | 1992 | 3 | 2 | 0 | (0, 0.56) |
|  | 1996 | 2 | 2 | 0 | (0, 0.66) |
|  | 1997 | 1 | 1 | 0 | (0, 0.79) |
|  | 1998 | 3 | 1 | 0 | (0, 0.56) |
|  | 1999 | 2 | 1 | 1 | (0.34, 1) |
|  | 2003 | 7 | 1 | 0 | (0, 0.35) |
|  | 2004 | 7 | 3 | 0 | (0, 0.35) |
|  | 2005 | 3 | 2 | 1 | (0.44, 1) |
| **NR Adults** | 1991 | 10 | 2 | 0.60 | (0.31, 0.83) |
|  | 1992 | 7 | 2 | 0.57 | (0.25, 0.84) |
|  | 1996 | 9 | 2 | 0.33 | (0.12, 0.65) |
|  | 1997 | 5 | 1 | 0.60 | (0.23, 0.88) |
|  | 1998 | 4 | 1 | 0 | (0, 0.49) |
|  | 1999 | 5 | 1 | 1 | (0.57, 1) |
|  | 2003 | 5 | 1 | 0 | (0, 0.43) |
|  | 2004 | 16 | 3 | 0.06 | (0.01, 0.28) |
|  | 2005 | 8 | 3 | 0.88 | (0.53, 0.98) |

Sample sizes and the number of packs (for wolves) or regions (for coyotes) sampled are noted.
