# Supplementary figures and images for: A Serological Survey of Infectious Disease in Yellowstone National Park’s Canid Community
Source: PLoS One. 2009 Sep 16;4(9):e7042. doi: 10.1371/journal.pone.0007042 (PMC2738425; doi:10.1371/journal.pone.0007042)

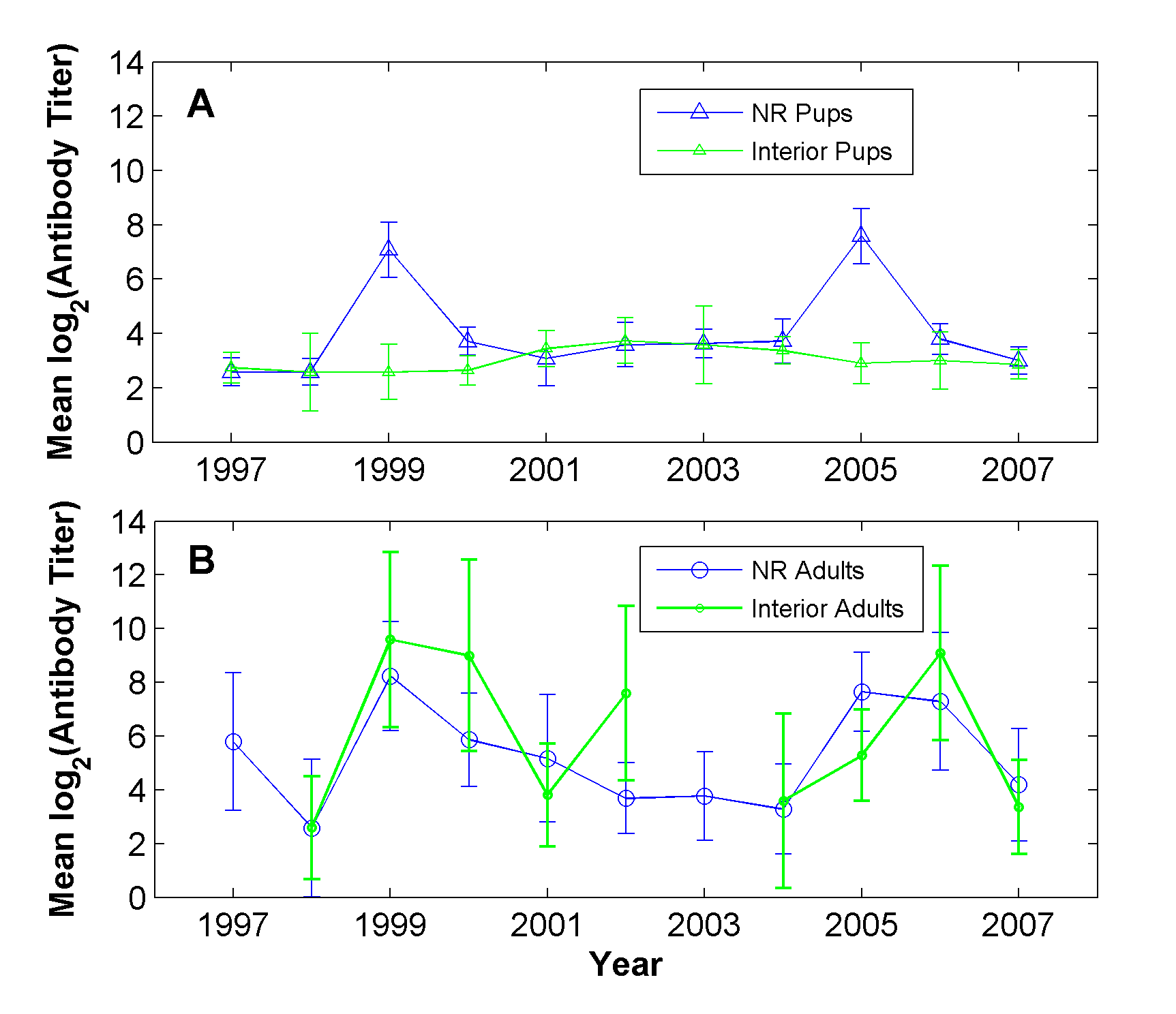

Supplement: Figure S1 — Mean wolf antibody titers to canine distemper virus in Yellowstone National Park, 1997–2007. Mean log2(antibody titers) are displayed with corresponding 95% confidence intervals for Northern Range (NR) and Interior pups (A) and adults (B). (7.71 MB TIF) [file pone.0007042.s004.tif]
